# Effects of Maternal Pterostilbene Supplementation on Milk Composition and Offspring Gut Antioxidant/Lipid Metabolism in Suckling Piglets: A Multi-Omics Study

**DOI:** 10.3390/antiox15050531

**Published:** 2026-04-23

**Authors:** Liyun Bai, Jiaqi Dong, Mingming Cao, Jiajun Hao, Houyu Jin, Zhongyu Li, Baoming Shi, Haoyang Sun, Xiao Liu

**Affiliations:** College of Animal Science and Technology, Northeast Agricultural University, Harbin 150030, China

**Keywords:** intestinal barrier, colonic mucosa, milk microbiota, metabolomics, suckling piglets, pterostilbene

## Abstract

This study aimed to investigate the effects of pterostilbene (PTE) on the intestinal barrier function, antioxidant capacity, lipid metabolism, and microbial and metabolite homeostasis of suckling piglets via its action on breast milk. Findings indicate that PTE supplementation enhanced the antioxidant status of mature milk and strengthened intestinal barrier function in piglets. Specifically, PTE enhanced intestinal antioxidant status and fatty acid β-oxidation in piglets by regulating the PI3K-AKT and SIRT1-Nrf2/Keap1 signaling pathways. 16S rDNA sequencing and Liquid Chromatography–Mass Spectroscopy (LC–MS) identified breast milk and gut microbiota and their metabolites, respectively. Results indicate that PTE significantly elevated levels of amino acid derivatives in colostrum (Glutathione Reducedform (GSH) and N-acetyl-L-glutamate (NAG)), whilst concurrently reducing levels of glycerophospholipid-related metabolites in both colostrum and mature milk (*p* < 0.05). Moreover, PTE supplementation markedly altered the composition of the colonic mucosal microbiota in piglets, with *Faecalibacterium*, *Mucispirillum* and *Ruminococcus* identified as key beneficial microbial markers of the colonic mucosa. Combined multi-omics revealed strong correlations in microbial community composition between mature milk and the colon, identifying glycerophospholipid metabolism as a key metabolic pathway that may be associated with the regulatory effects of PTE on milk and the piglet colon. In conclusion, the PTE supplement can improve the quality of breast milk and have a positive impact on the intestinal homeostasis of the offspring.

## 1. Introduction

As the sole food source during lactation, breast milk provides the best nutritional support for the growth and development of the offspring. Breast milk is rich in nutrients, immunomodulators, and microbial communities, contributing to the coordinated development of the offspring’s intestinal ecosystem and exerting profound impacts on both short- and long-term health. Lactating mothers are in a state of high catabolism and experience persistent oxidative stress; this may lead to alterations in the nutritional composition of breast milk, thereby affecting the developmental trajectory and overall health of the nursing offspring [1]. Maternal diet, as a key regulatory factor, can reshape the nutritional composition and microbial community structure of breast milk, thereby improving offspring gut health [2]. The exogenous nutritional supplementation of lactating mothers is receiving increasing attention because of growing evidence that specific dietary and nutritional patterns are associated with health benefits for mothers and infants. Dietary supplementation with natural phytochemicals during lactation offers nutritional benefits. It enhances the antioxidant capacity of breast milk, promotes the translocation of beneficial bacteria, and strengthens the intestinal barrier, thereby contributing to the early establishment and long-term maintenance of intestinal homeostasis in newborns [3,4].

Among these, polyphenolic compounds exhibit remarkable biological activities with favorable biosafety, positioning them as promising therapeutic candidates [5]. PTE is a natural trans-stilbene compound. It was initially isolated from *pterocarpus santalinus* plants [6] and demonstrates superior pharmacokinetic properties compared to its analog resveratrol (RSV). Structural modifications (methoxy substitutions at positions 3 and 5) enhance PTE’s lipophilicity, oral bioavailability, and chemical stability [7]. Previous studies conducted by our laboratory have demonstrated that PTE activates the PI3K-AKT-mTOR pathway to alleviate oxidative stress in sows and effectively promote nutrient transport in the placenta [8]. Animal studies have demonstrated that PTE activates the nuclear factor erythroid 2-related factor 2 (Nrf2) pathway and ameliorates intestinal injury in weaned piglets with intrauterine growth restriction (IUGR), while also attenuating oxidative tissue damage through a sirtuin 1 (SIRT1)-mediated pathway [9,10]. In addition, there are also studies that show PTE’s capacity to remodel gut microbial communities, ameliorate obesity-associated metabolic dysregulation, and restore energy homeostasis [11].

Although the beneficial effects of maternal dietary polyphenol supplementation on offspring intestinal health have been well-established, the underlying mechanisms—particularly the regulatory role of PTE supplementation mediated via the “breast milk microbiota-metabolite” axis on the “microbiota-metabolite” axis in the offspring’s intestine—remain largely unexplored. This study hypothesizes that maternal dietary supplementation with PTE can specifically alter the composition of breast milk microbiota and its metabolites, thereby reshaping the intestinal microbial ecosystem and metabolic functions in piglets. Ultimately, this may improve the redox homeostasis and metabolic balance in the offspring’s gut, enhancing the intestinal barrier function of piglets. To validate this hypothesis, this study used lactating sows and their suckling piglets as a model. This study employs integrated multi-omics analysis as a key approach to investigating how PTE modulates intestinal health in piglets by mediating the effects of breast milk at the microbial–metabolite interaction level. This provides a theoretical basis for optimizing early gut health in offspring through maternal dietary supplementation with functional food components.

## 2. Materials and Methods

### 2.1. Animals and Diets

In this experiment, single-factor randomized experimental design was used to select 12 healthy, close-weight pregnant Landrace × Large White hybrid sows with 2 to 4 litters. All sows were randomly divided into two groups of 6 replicates each. The control group was fed a basal diet, and the PTE group accepted a basal diet supplemented with 500 mg/kg PTE (PTE, purity: 97%; Shanghai Yuanye Biological Co., Ltd., Shanghai, China). The randomization sequence was generated using the standard =RAND() function in Microsoft Excel. The choice of PTE concentration was based on the results of a previous study in our laboratory [8]. The trial started from the first 7 days before parturition and was maintained until the piglets were weaned at 21 days. The experimental sows were housed in farrowing crates measuring 0.7 m × 2.2 m, and the crates were completely randomized in their placement to eliminate location bias. The ambient temperature in the farrowing room was controlled at 20–24 °C, with a relative humidity of 55–65%. Piglet areas were provided with local heating via heat lamps, and the temperature was adjusted according to the ambient temperature in the farrowing room to ensure a comfortable thermal environment for piglets. Piglet creep boxes were heated from 1 day before the expected farrowing date until piglets were weaned at 21 days of age. All sows and piglets had free access to clean drinking water throughout the experimental period. During the entire lactation period, no creep feed was provided to the piglets, ensuring that sow milk was the sole nutritional source for the piglets. To standardize nutritional intake and minimize litter-to-litter variation, cross-fostering was performed within 24 h after birth to standardize litter size per sow and ensure an even distribution of initial body weight among piglets. Piglets had unrestricted access to their sows’ teats and were allowed to nurse freely until the conclusion of the experiment. One piglet per litter from each treatment group was selected for subsequent experiments. The sows were from the location used for this trial: Fujin Farm in Jiamusi. The gestation and lactation diets (Table S1) were formulated to meet the nutritional recommendations of the NRC (2012) [12].

### 2.2. Sample Collection and Preparation

The sow’s anterior, middle, posterior, and hind teats were used to collect 15–25 mL of breast milk (both colostrum and mature milk) on the day of parturition and weaning. The colostrum was separated into control (UC) and experimental (UP) groups, and the mature milk was separated into control (AC) and experimental (AP) groups. Each treatment group collected a total of 6 litters of piglets. One piglet per litter, with a body weight approximately equal to the litter average, was randomly selected and euthanized with sodium pentobarbital, and tissue samples were subsequently collected to analyze the anatomical characteristics of 21-day-old suckling piglets. After the pigs were slaughtered, the intact gastrointestinal tract was removed, and the jejunum and colon were collected sequentially. A portion of the jejunum and colon were removed, the piglet colon and jejunal tissue samples were rinsed with saline, and the contents were removed under saline rinsing; then, the intestinal mucosa was scraped with a sterile slide and loaded into 2 mL sterile freezer tubes. A small portion of the samples was preserved in 4% paraformaldehyde solution, and the remaining samples were transferred to pre-labelled cryopreservation tubes and snap-frozen in liquid nitrogen. These samples were then stored at −80 °C, with the fixated samples kept at −20 °C. Follow-up assessments will include examining the antioxidant capacity of breast milk, its microbiota, and their metabolites. Additionally, evaluations took place of the intestinal tissue morphology, structural proteins, antioxidant levels, lipid metabolism, microbiota, and their metabolites within intestinal tissues.

### 2.3. Growth Performance

Within 24 h after farrowing, each newborn piglet was individually weighed to record its birth weight, and the total litter birth weight was calculated (pre-fostering litter weight). After cross-fostering, all piglets in the reconstituted litters were individually weighed again to obtain the post-fostering litter weight as the starting weight for growth performance evaluation. At weaning (21 days of age), each piglet was individually weighed, and the total litter weaning weight was recorded. Using the individual body weights collected within 24 h after farrowing and at weaning, the following growth performance indicators were calculated for each litter: total live litter weight at birth, average live weight at birth, total litter weight at weaning, average weight at weaning, litter weight gain, and average daily gain (ADG).

### 2.4. Histopathological Observations of the Intestinal Tract

The jejunum and colon tissues were fixed in 4% paraformaldehyde for 24 h, then dehydrated, dipped in wax, embedded, sectioned, deparaffinized, stained, and sealed, and the tissue sections were placed under observation and photographed. The height of jejunum villi/depth of crypt (V/C) as well as the crypt depth of colon were counted using ImageJ (version 1.54g, NIH, Bethesda, MD, USA) software. All measurements were normalized for processing.

### 2.5. Immunofluorescence Examination

Paraffin-embedded sections of the jejunum and colon were prepared for IF staining of Claudin-1 and zonula Occludens-1 (ZO-1). Anti-Claudin-1 antibody (Abclonal, A2196) and Anti-ZO-1 antibody (Abclonal, A0659) were purchased from Abclonal (Wuhan, China). Three randomly selected fields of view for each produced slide were observed with a fluorescence microscope (EVOS M700, Thermo Fisher Scientific, Jena, Germany) and quantitatively analyzed.

### 2.6. Oxidative Stress Assessment

Breast milk, as well as the jejunum and colon of piglets, was analyzed for antioxidant indicators including total antioxidant capacity (T-AOC), malondialdehyde (MDA), superoxide dismutase (SOD), glutathione peroxidase (GSH-Px), and catalase (CAT). Commercial kits purchased from Suzhou Grace Biotechnology (Suzhou, China) were used strictly according to the manufacturer’s instructions.

### 2.7. Quantitative Real-Time PCR Analysis

A total of 0.1 g of intestinal tissue was accurately weighed, and 1 mL of TRIzol reagent was added to each sample to extract total RNA, which was solubilized by 4 degrees of milling, lysis, and, finally, adding diethyl pyrocarbonate-treated water. Total RNA concentration and purity were determined by an ultramicro-spectrophotometer (Implen GmbH, Munich, Germany). The minimum concentration of total RNA was greater than 500 ng/µL, and the ratio of A260/A280 ranged from 1.8 to 2.0. Eligible RNA was reverse transcribed into cDNA by 5× Integrated RT MasterMix according to the manufacturer’s instructions (DiNing, Beijing, China). The qRT-PCR was performed strictly according to the instructions (Takara, Dalian, China). The designed and synthesized qRT-PCR primers are listed in Table S2. The primers used were queried on the official NCBI website, and the results were presented as 2^−ΔΔCt^ to demonstrate the content.

### 2.8. Western Blotting

Intestinal tissues (0.2 g) were weighed in 1 mL of radioimmunoprecipitation assay lysis solution (Beyotime, Shanghai, China) and supplemented with protease inhibitor and phosphatase inhibitor solutions. The lysed samples were centrifuged at 4 °C, 12,000 rpm for 15 min, and the supernatant was taken to determine the protein content using the BCA Protein Assay Kit (Epizyme Biotech, Shanghai, China). Sample proteins were separated by SDS-PAGE electrophoresis and transferred to a 0.45 μm polyvinylidene difluoride (PVDF) membrane activated by anhydrous ethanol, which were rinsed with TBST for 5 min and incubated with a 1-fold dilution of the blocking solution (Epizyme Biotech, Shanghai, China) for 10 min. The diluted primary antibody was added to the antibody incubator and incubated for 12 h at 4 °C in a refrigerator. On the next day, protein-bound PVDF membranes were washed five times with 1× TBST, incubated with secondary antibody for one hour, and washed five more times. Antibody-tagged proteins were visualized by a gel imaging and analysis system (Alpha Innotech Corporation, San Leandro, CA, USA) and quantified by ImageJ (version 1.54g, NIH, Bethesda, MD, USA). β-actin was used for normalization. After fluorescence detection, the PVDF membrane was rinsed with TBST and incubated in stripping buffer (YaEnzyme, Shanghai, China) to remove bound antibodies. The membrane was then thoroughly washed with TBST, followed by re-blocking and proceeding with further antibody incubations. Refer to Table S3 for the antibodies used in this study.

### 2.9. Bioinformatics Analysis of 16S rDNA Gene Sequencing

According to the manufacturer’s instructions, total bacterial DNA was extracted from breast milk and colonic mucosa samples using the CTAB method. The V3–V4 hypervariable region of the 16S rRNA gene was amplified via PCR. The constructed libraries were sequenced on the Illumina NovaSeq platform, and raw reads were filtered to obtain high-quality Clean Tags. To resolve exact biological sequences, Amplicon Sequence Variants (ASVs) were generated using the Deblur (v1.1.1) denoising algorithm. The taxonomic assignment of representative ASV sequences was performed using the Mothur (v1.48) classifier against the SILVA 138.1 reference database. Alpha and beta diversity analyses were conducted in R software (v4.3.2) utilizing the vegan (v2.6.4) and phyloseq (v1.46.0) packages to characterize microbial community structures. Ordination plots including principal component analysis (PCA) and principal coordinate analysis (PCoA) were generated using ggplot2 (v3.5.0). Significantly differential taxa between groups were identified using linear discriminant analysis (LDA) effect size (LEfSe) analysis (v1.1.2). The functional potential of the microbial communities was predicted using Tax4Fun2 (v1.1.5). The raw 16S rDNA sequencing data has been deposited in the NCBI Sequence Read Library under accession number PRJNA1430079 and can be accessed directly at https://www.ncbi.nlm.nih.gov/bioproject/PRJNA1430079 (accessed on 12 April 2026). Outlier detection was performed based on the Bray–Curtis distance matrix. A sample was defined as an outlier and excluded if its average within-group distance exceeded the mean distance of the group plus two standard deviations (Mean + 2SD), indicating significant deviation from other biological replicates. PCoA identified one outlier in the sow colostrum samples of each group. To ensure sample pairing consistency across multi-omics data, all corresponding samples from this sow, including mature milk as well as gut microbial 16S data of the piglets suckled by this sow, were excluded synchronously. Consequently, the final sample size for 16S rRNA gene sequencing analysis was five samples per group.

### 2.10. Metabolomics Analysis

Breast milk and colonic mucosa tissue samples were thawed on ice and transferred into corresponding numbered centrifuge tubes with corresponding volumes of mixed extracts of acetonitrile, methanol and water, then centrifuged at 12,000 rpm for 15 min at 4 °C, and the supernatants were pipetted for subsequent analysis. Both samples were analyzed by Liquid Chromatography–Mass Spectroscopy (LC–MS), and mass spectral peaks were extracted, corrected, and filtered for metabolite identification and annotation. The data acquisition was operated using the information-dependent acquisition (IDA) mode using Analyst TF 1.7.1 Software (Sciex, Concord, ON, Canada). Metabolites with an identification composite score ≥ 0.5 and a CV value < 0.3 were selected as QC samples, followed by merging positive and negative ion modes to obtain high-quality data. All statistical analyses were performed using R software (v4.1.2). PCA was conducted using the R base package. Orthogonal Partial Least Squares Discriminant Analysis (OPLS-DA) was carried out using the MetaboAnalystR package (v1.0.1) following log_2_ transformation and mean centering. Heatmaps were generated using the ComplexHeatmap package (v2.9.4). Sample correlation analysis was performed using Spearman’s rank correlation and visualized with the corrplot package (v0.92). Correlation networks were constructed using the igraph package (v1.2.11) and ggraph package (v2.0.5). Metabolites meeting the criteria of VIP > 1, *p* < 0.05, and |log_2_FC| ≥ 1 were considered significantly differential. The metabolic pathway enrichment analysis of the identified metabolites was performed using MetaboAnalyst 6.0 with reference to the KEGG and HMDB databases [13]. Based on the sample exclusion results from 16S rDNA gene sequencing, matched metabolomics samples were also excluded to maintain a strict one-to-one correspondence within the multi-omics dataset and ensure the statistical validity of the paired analysis. The final sample size for the metabolomics analysis was five samples per group.

### 2.11. Correlation Analysis of Metabolites and Bacteria

Spearman’s correlation analysis was performed to determine the associations between differentially expressed metabolites and differential microbial taxa. Correlation analyses were calculated using the cor function of the R software and significance tests for correlations were obtained using the corPvalueStudent function of the WGCNA of the R software to generate correlation heat maps.

### 2.12. Statistical Analysis of Data

All data were analyzed using SPSS Statistics Software (version 27.0, IBM Corp., Armonk, NY, USA). For data satisfying the assumptions of normality and homogeneity of variance, a two-tailed independent samples *t*-test was used for analysis. For data failing to meet these assumptions, the nonparametric Mann–Whitney U test was employed instead. Statistics, calculations and the categorization of raw data were performed through Microsoft Excel. Data obtained are expressed as ±standard error of the mean; ** *p* < 0.01 indicates a highly significant difference, and * *p* < 0.05 indicates a significant difference between groups. Results were plotted using GraphPadPrism (version 9.5.1, GraphPad Software, New York, NY, USA).

## 3. Results

### 3.1. The Effect of PTE on Piglet Production Performance

As shown in Table 1, compared with the control group, the addition of PTE had no significant effect on total live litter weight at birth, average live weight at birth, total litter weight at weaning, average weight at weaning, litter weight gain, or ADG.

### 3.2. Effect of PTE on the Antioxidant Capacity of Sow’s Milk

**Figure 1:**
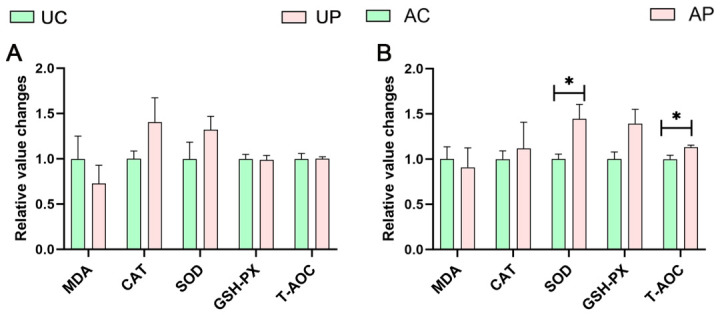
Effect of PTE on the antioxidant capacity of sow’s milk. (**A**) Antioxidant indicators in the colostrum. (**B**) Antioxidant indicators in mature milk. MDA, malondialdehyde; CAT, catalase; SOD, superoxide dismutase; GSH-Px, glutathione peroxidase; T-AOC, total Antioxidant Capacity. UC, AC: control group, UP, AP: pterostilbene group. Data are expressed as mean ± SEM (*n* = 6). * *p* < 0.05, compared with control group.

As shown in Figure 1A, the UP group exhibited no significant effect on any antioxidant indicators compared with the UC group. However, Figure 1B indicates that the levels of SOD and T-AOC in the AP group were significantly higher than those in the AC group (*p* < 0.05), suggesting enhanced antioxidant capacity in mature milk.

### 3.3. Effect of PTE on Colostrum Microbiota in Sows

**Figure 2:**
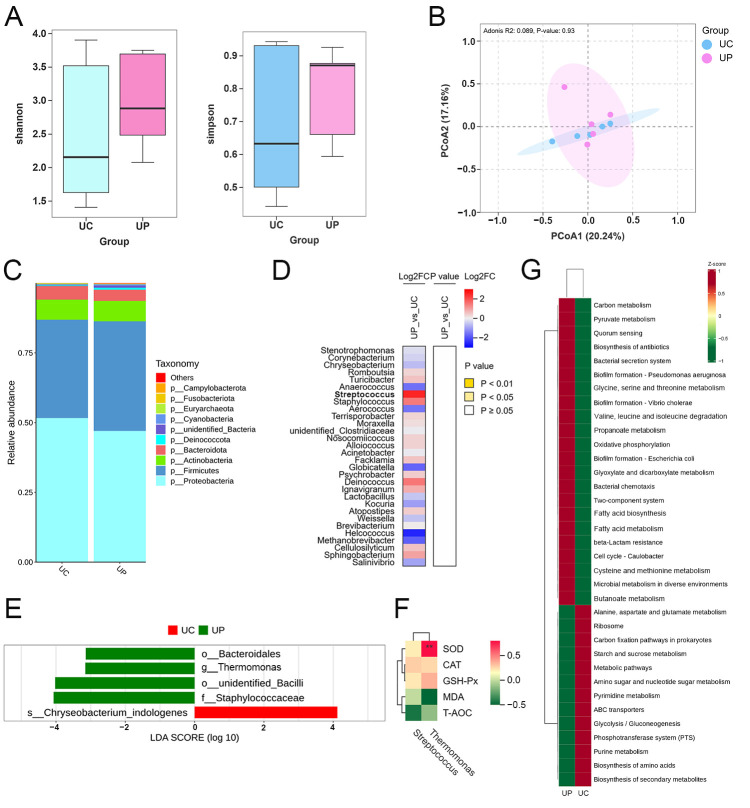
Effect of PTE on colostrum microbiota in sows. (**A**) Colostrum microbiota alpha diversity (Shannon and Simpson). (**B**) Colostrum microbiota beta diversity. (**C**) Relative abundance at the phylum level of the microbiota. (**D**) Heat map of genus-level abundance of the top-30 microbiota. (**E**) LEfSe analysis. (**F**) Spearman correlation analysis of dominant genera with antioxidant indications. (**G**) Tax4Fun2 microbial function prediction. UC: control group; UP: pterostilbene group. ** *p* < 0.01, compared with control group (*n* = 5).

In order to investigate the potential mechanisms by which the maternal dietary addition of PTE regulates milk quality, we analyzed the effect of PTE on the microbial composition of sow colostrum by 16S rDNA. As shown in Figure 2A,B, the addition of PTE had no significant effect on the α-diversity and β-diversity of microbiota in sow colostrum in this study. Figure 2C displays that the colostrum microbiota at the phylum level was dominated by Proteobacteria (UC: 43.07%, UP: 39.20%) and Firmicutes (UC: 29.31%, UP: 32.75%). The heatmap of the top-30 genera shows that *Streptococcus* exhibited the most pronounced variation in relative abundance across groups (FC = 5.82), although no statistically significant differences were observed (Figure 2D). Individual contributions of differential taxa to the UP group were determined using LEfSe, and, as shown in Figure 2E, *Thermomonas* was the dominant genus in the UP group (LDA > 3). Spearman correlation analysis revealed a significant positive correlation between *Thermomonas* and SOD (Figure 2F). The functional prediction of the colostrum microbial community was performed using Tax4Fun2 (Figure 2G). It was found that the UP group was mostly enriched in amino acid-related pathways (glycine, serine and threonine metabolism; valine, leucine and isoleucine degradation; and cysteine and methionine metabolism) and lipid metabolism-related pathways (butanoate metabolism, propanoate metabolism, fatty acid biosynthesis, and fatty acid metabolism).

### 3.4. Effect of PTE on Colostrum Metabolites in Sows

**Figure 3:**
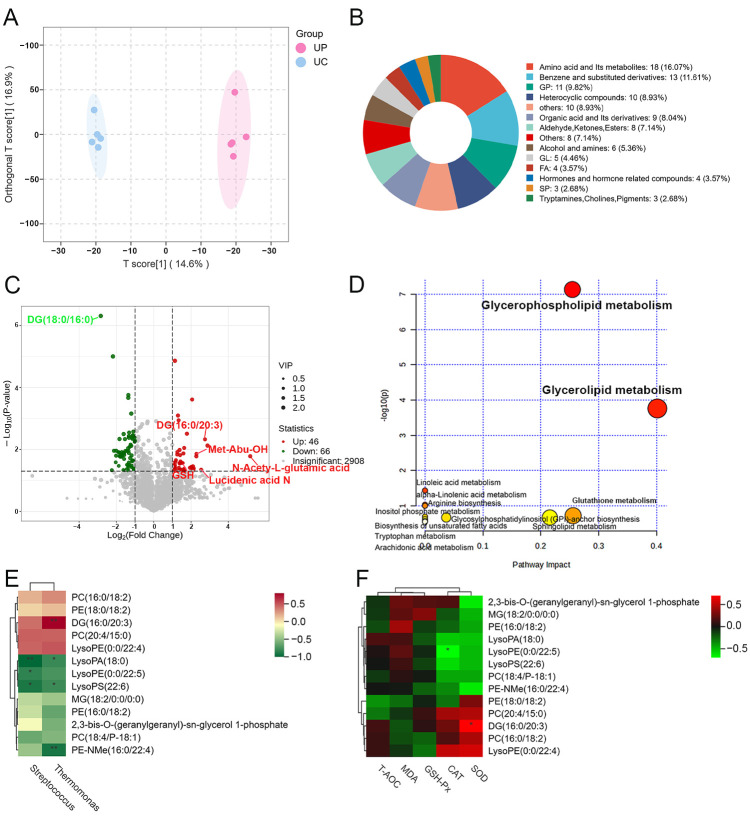
Effect of PTE on colostrum metabolites in sows. (**A**) OPLS-DA analysis of metabolite composition. (**B**) Differential metabolite classification map. (**C**) Metabolite volcano plot (Red dots indicate significantly upregulated metabolites, and green dots indicate significantly downregulated metabolites). (**D**) Metabolic pathways according to the MetaboAnalyst analysis. (**E**) Correlations between different bacterial genera and different metabolites based on Spearman analysis. (**F**) Correlations between different metabolites and antioxidant indices based on Spearman analysis. UC: control group; UP: pterostilbene group. * *p* < 0.05 and ** *p* < 0.01, compared with control group (*n* = 5).

An untargeted metabolomics technique was used to learn more about how adding PTE to the sow diet affected the metabolism of colostrum. Detailed information on all metabolites identified in colostrum is provided in Table S4. OPLS-DA results (Figure 3A) showed a significant separation of metabolites in both UC and UP groups, demonstrating that PTE significantly altered the metabolites of colostrum. According to the OPLS-DA model, the differential metabolites were screened according to the conditions of VIP > 1, *p* < 0.05, and |log2FC| > 1. Among them, Figure 3B displays the top-five classes of primary differential expressed metabolites (DEMs): amino acids and their metabolites (16.07%), glycerophospholipids (GP) (9.82%), organic acids and derivatives (8.04%), glycolipids (GL) (4.46%), and fatty acids (FAs) (3.57%). The volcano plot demonstrated the trend of differential metabolites in the two groups, with a total of 112 significant differential metabolites, 46 upregulated and 66 downregulated (Figure 3C). The top-five primary DEMs ranked by Log2FC value include DG(18:0/16:0), DG (16:0/20:3), Met-Abu-OH, N-Acety-L-glutamic acid and Lucidenic acid N. It is notable that the level of GSH, which has a strong antioxidant function, has also significantly increased. Based on the 112 differential metabolites identified, MetaboAnalyst was used to link the metabolomics data obtained to potential biochemical pathway regulation (Figure 3D). Twelve metabolic pathways were enriched, containing mainly three major amino acid metabolic pathways as well as seven lipid metabolic pathways. Only two lipid metabolism pathways (glycerophospholipid metabolism and glycerolipid metabolism) remained significant after false discovery rate (FDR) correction (Benjamini–Hochberg method, q < 0.05). Further, the performed Spearman correlation analysis between the significantly differential metabolites showed enrichment in these two key metabolic pathways and the dominant bacterial genera (at the genus level) (Figure 3E). *Streptococcus* showed a negative correlation with lysophosphatidic acid (18:0) [LysoPA (18:0)], lysophosphatidylserine (22:6) [LysoPS (22:6)] and lysophosphatidylethanolamine (0:0/22:5) [LysoPE (0:0/22:5)]. Additionally, there was a negative correlation between *Thermomonas* and LysoPA (18:0), LysoPS (22:6), and Phosphorylethanolamine-N-methyl (16:0/22:4) [PE-NMe (16:0/22:4)]. In contrast, *Thermomonas* was significantly positively correlated with DG (16:0/20:3). Furthermore, the correlation between colostrum differential metabolites and their antioxidant indication was further analyzed (Figure 3F), we found that DG (16:0/20:3) showed a significant positive correlation with SOD. The above metabolite may serve as a potential biomarker reflecting the antioxidant capacity of colostrum. In conclusion, the addition of PTE to the maternal diet significantly remodeled lipid metabolism in colostrum, particularly within the glycerophospholipid metabolism and glycerolipid metabolism.

### 3.5. Effect of PTE on the Microbiota of Mature Milk in Sows

**Figure 4:**
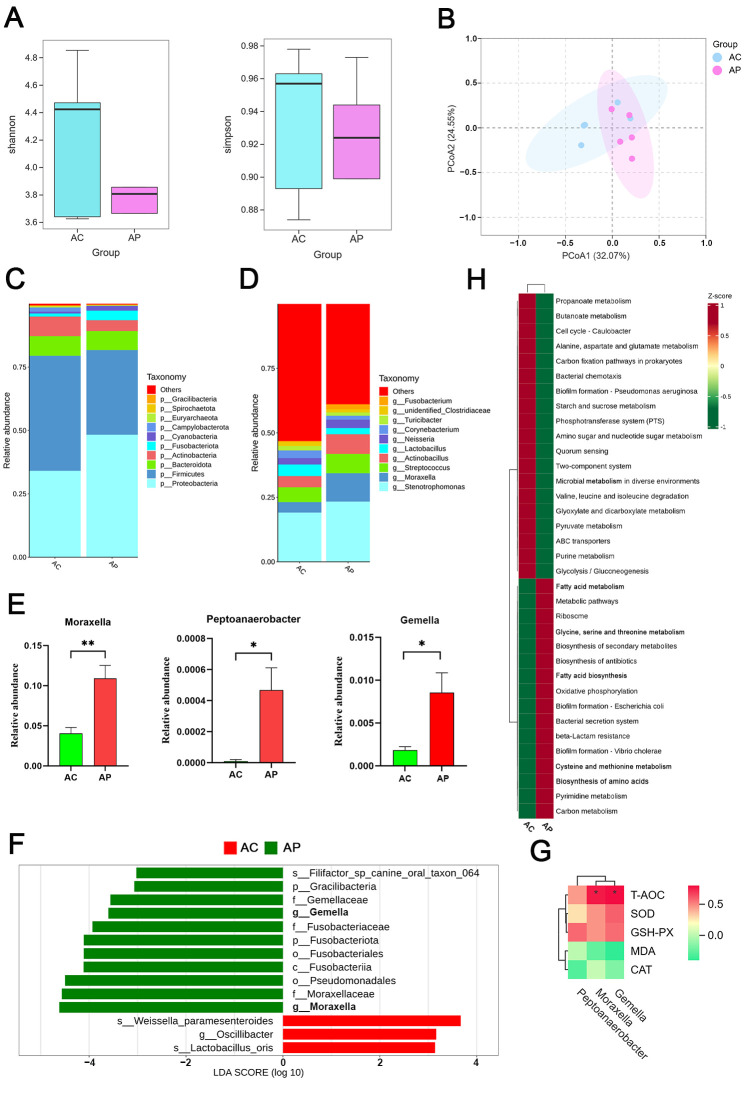
Effect of PTE on the microbiota of mature milk in sows. (**A**) Mature milk microbiota alpha diversity (Shannon and Simpson). (**B**) Mature milk microbiota beta diversity. (**C**,**D**) Relative abundance at the microbial phylum and genus level. (**E**) *t*-test for microbial genus level of mature milk. (**F**) LEfSe analysis. (**G**) Spearman correlation analysis of dominant genera with antioxidant indications. (**H**) Tax4Fun2 microbial function prediction. AC: control group; AP: pterostilbene group. * *p* < 0.05 and ** p < 0.01, compared with control group (*n* = 5).

As shown in Figure 4A,B, the present study found that the addition of PTE did not significantly affect the α-diversity and β-diversity of the mature milk microbiota of sows. The different distributions of the mature milk microbiota were analyzed at the phylum and genus levels (Figure 4C–E). Figure 4C displays that the mature milk microbiota at the phylum level was dominated by Proteobacteria (AC: 28.40%, AP: 40.22%) and Firmicutes (AC: 37.76%, AP: 27.87%). Among the identified genera, the relative abundances of *Peptoanaerobacter* (AC: 1.03 × 10^−5^ vs. AP: 0.00047), *Gemella* (AC: 0.00184 vs. AP: 0.00856), and *Moraxella* (AC: 0.04058 vs. AP: 0.10902) were significantly higher in the AP group than in the AC group (Figure 4D,E *p* < 0.05). LEfSe analyses also demonstrated that *Gemella* and *Moraxella* were the dominant genera in the AP group (Figure 4F, LDA > 3). Spearman correlation analysis revealed significant positive associations between both *Gemella* and *Moraxella* with T-AOC (Figure 4G). The functional prediction of the mature milk microbial community was performed using Tax4Fun2 (Figure 4H). It was found that the AP group was mostly enriched in amino acid-related pathways (glycine, serine and threonine metabolism; cysteine and methionine metabolism; and biosynthesis of amino acids) and lipid metabolism-related pathways (fatty acid biosynthesis and fatty acid metabolism).

### 3.6. Effect of PTE on Metabolites of Mature Milk in Sows

**Figure 5:**
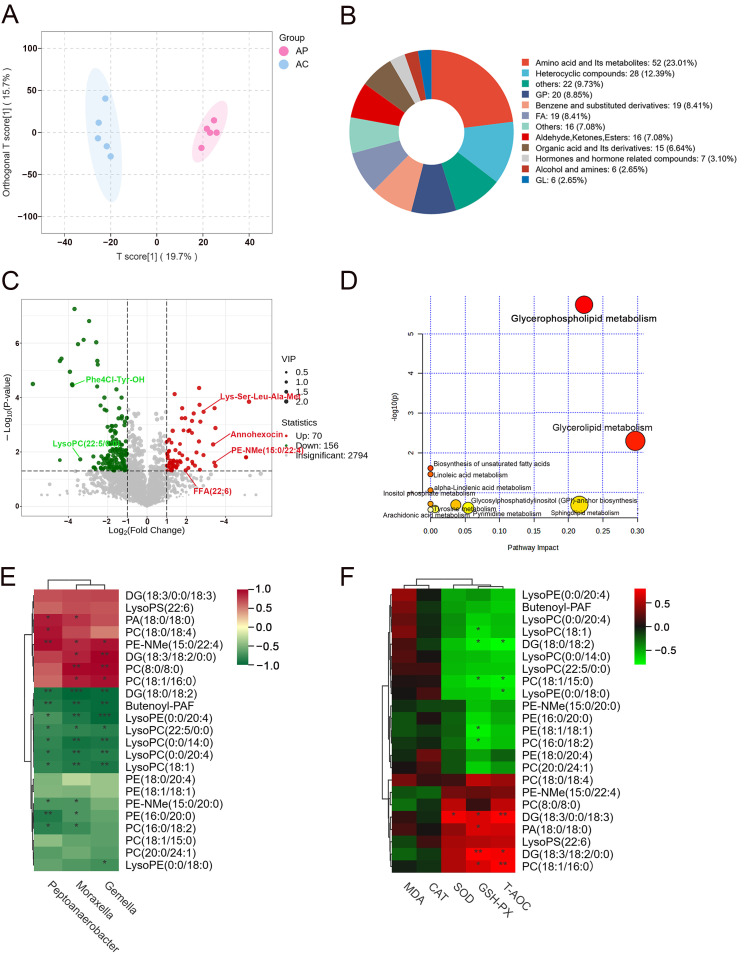
Effect of PTE on mature milk metabolites in sows. (**A**) OPLS-DA analysis of metabolite composition. (**B**) Differential metabolite classification map. (**C**) Metabolite volcano plot (Red dots indicate significantly upregulated metabolites, and green dots indicate significantly downregulated metabolites). (**D**) Metabolic pathways according to the MetaboAnalyst analysis. (**E**) Correlations between different bacterial genera and different metabolites based on Spearman analysis. (**F**) Correlations between different metabolites and antioxidant indices based on Spearman analysis. AC: control group; AP: pterostilbene group. * *p* < 0.05, ** p < 0.01 and *** p < 0.001, compared with control group (*n* = 5).

An untargeted metabolomics technique was further employed to assess the levels of mature milk metabolites. Detailed information on all metabolites identified in mature milk is provided in Table S5. OPLS-DA results showed that there was a significant separation of metabolites in both the AC and AP groups (Figure 5A). Differential metabolites were screened under the same conditions according to the OPLS-DA model. Among them, Figure 5B displays the top-five classes of primary DEMs: amino acid and its metabolites (23.01%), GP (8.85%), FA (8.41%), organic acid and its metabolites (6.64%) and GL (2.65%). The volcano plot demonstrated the trend of differential metabolites in the two groups, with a total of 226 significant differential metabolites, 156 downregulated and 70 upregulated (Figure 5C). The top-five primary DEMs ranked by Log2FC value include Lys-Ser-Leu-Ala-Met, Ph4Cl-Try-OH, PE-NMe (15:0/22:4), LysoPC (22:5/0:0) and Annohexocin. However, it is worth noting that the level of FFA (22:6) in the AP group significantly increased. Based on the 226 differential metabolites identified, MetaboAnalyst was used to link the metabolomics data obtained to potential biochemical pathway regulation (Figure 5D). Eleven metabolic pathways were enriched, which mainly contained one amino acid metabolic pathway as well as seven lipid metabolic pathways. Among these, only one lipid metabolism pathway (glycerophospholipid metabolism) remained significant after FDR correction (Benjamini–Hochberg method, q < 0.05). Spearman correlation analysis was conducted on the differential metabolites enriched in this key metabolic pathway and the differential bacterial genera in mature milk to determine the correlations between them (Figure 5E). We could find that DG (18:3/18:2/0:0), Phosphatidylcholine [PC (8:0/8:0)] and PC (18:1/16:0) were positively correlated with *Gemella* and *Moraxella*. Phosphatidic acid [PA (18:0/18:0)] was positively correlated with *Moraxella* and *Peptoanaerobacter*. PC (18:0/18:4) was positively correlated with *Peptoanaerobacter*. PE-NMe (15:0/22:4) was positively correlated with all different genera. In contrast, LysoPE (0:0/20:4), Butenoyl-PAF, DG (18:0/18:2), LysoPC (0:0/14:0), LysoPC (18:1), LysoPC (0:0/20:4) and LysoPC (22:5/0:0) showed a negative correlation with all differential genera. In addition, the further analysis of the correlation between mature milk differential metabolites and their antioxidant indications revealed that DG (18:3/0:0/18:3) was positively correlated with SOD, GSH-Px and T-AOC. PA (18:0/18:0) was positively correlated with GSH-Px. PC (18:1/16:0) and DG (18:3/18:2/0:0) were positively correlated with GSH-Px and T-AOC (Figure 5F). The above metabolites may serve as potential biomarkers reflecting the antioxidant capacity of mature milk. In conclusion, the supplementation of PTE into the maternal diet reshaped the lipid metabolism in mature milk, particularly within the glycerophospholipid metabolism.

### 3.7. Effect of PTE on Intestinal Histomorphology and Mechanical Barriers in Piglets

**Figure 6:**
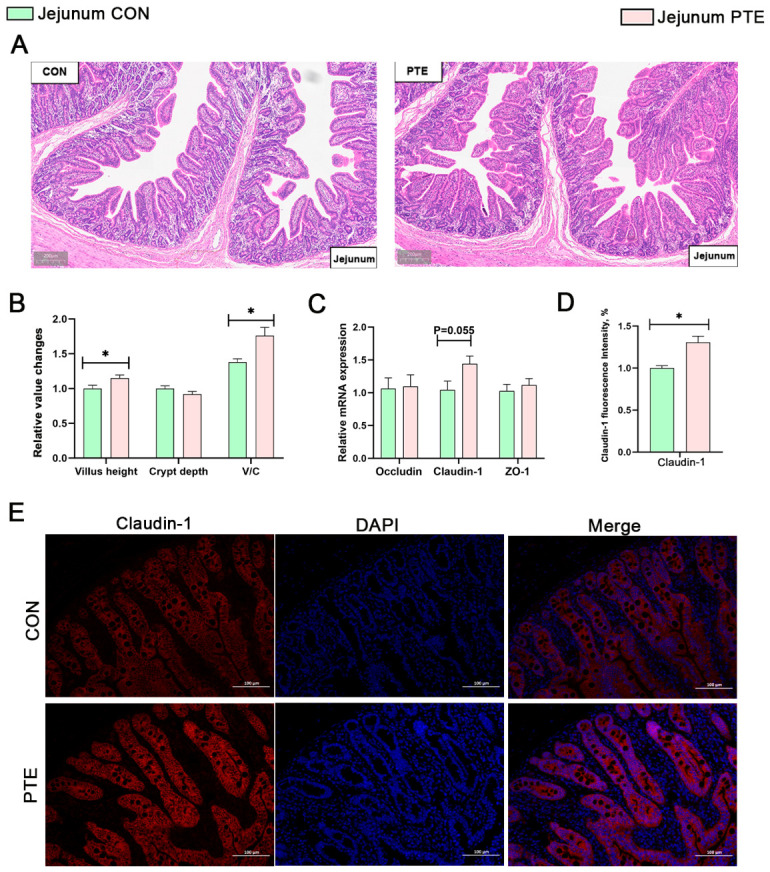
Effect of PTE on intestinal histomorphology and mechanical barriers in piglets. (**A**) HE staining results of the jejunum. (**B**) Jejunal morphological indices. (**C**) Intestinal structural protein mRNA expression. (**D**,**E**) Structural protein Claudin-1, 200× analysis of IF staining. CON: control group; PTE: pterostilbene group. Data are expressed as mean ± SEM (*n* = 6). * *p* < 0.05, compared with control group.

**Figure 7:**
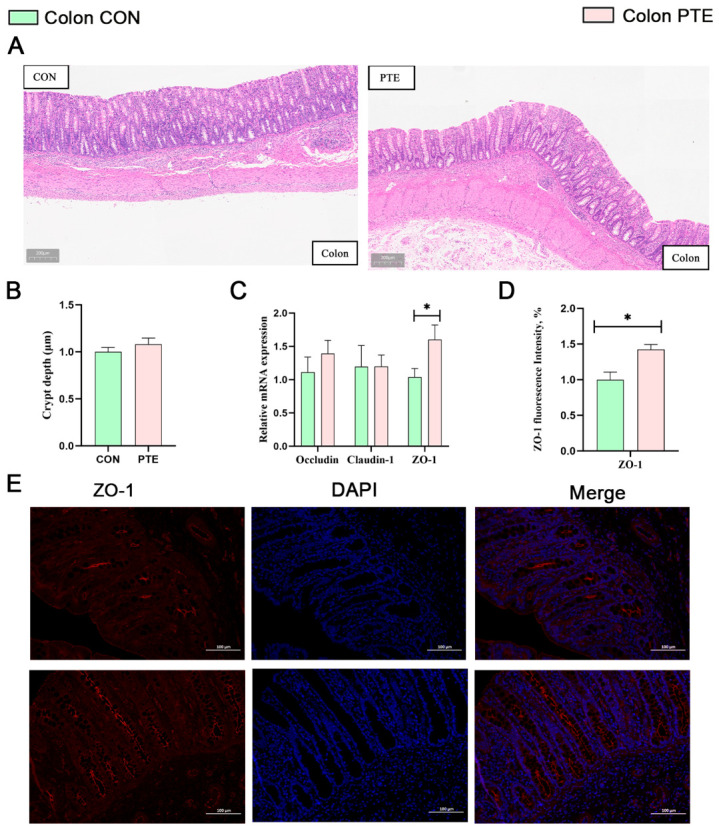
Effect of PTE on intestinal histomorphology and mechanical barriers in piglets. (**A**) HE staining results of the colon. (**B**) Colonic morphological indices. (**C**) Intestinal structural protein mRNA expression. (**D**,**E**) Structural protein ZO-1, 200× analysis of IF staining. CON: control group; PTE: pterostilbene group. Data are expressed as mean ± SEM (*n* = 6). * *p* < 0.05, compared with control group.

The villus–crypt ratio illustrates the small intestine’s capacity for digestion and absorption, whereas villus height (VH) and crypt depth (CD) are significant markers for evaluating intestinal health. The results of the HE staining of the jejunum were shown in Figure 6A,B. Compared with the CON group, the PTE group showed no significant effect on jejunal crypt depth in piglets, but the VH and the V/C ratio were significantly increased (*p* < 0.05). Among them, immunofluorescence staining demonstrated that PTE significantly promoted the protein expression of Claudin-1 (Figure 6D,E, *p* < 0.05), which was also confirmed by qRT-PCR analysis (Figure 6C, *p* = 0.055). The results of HE staining of the colon were shown in Figure 7A,B. There was no statistically significant difference in colonic crypts depth in the PTE group compared to the CON group. Among them, immunofluorescence staining demonstrated that PTE significantly promoted the protein expression of ZO-1 (Figure 7D,E, *p* < 0.05), which was also confirmed by qRT-PCR analysis (Figure 7C, *p* < 0.05).

### 3.8. Effect of PTE on SIRT1-Nrf2/Keap1 Pathway and Its Antioxidant Capacity in Piglet Intestine

**Figure 8:**
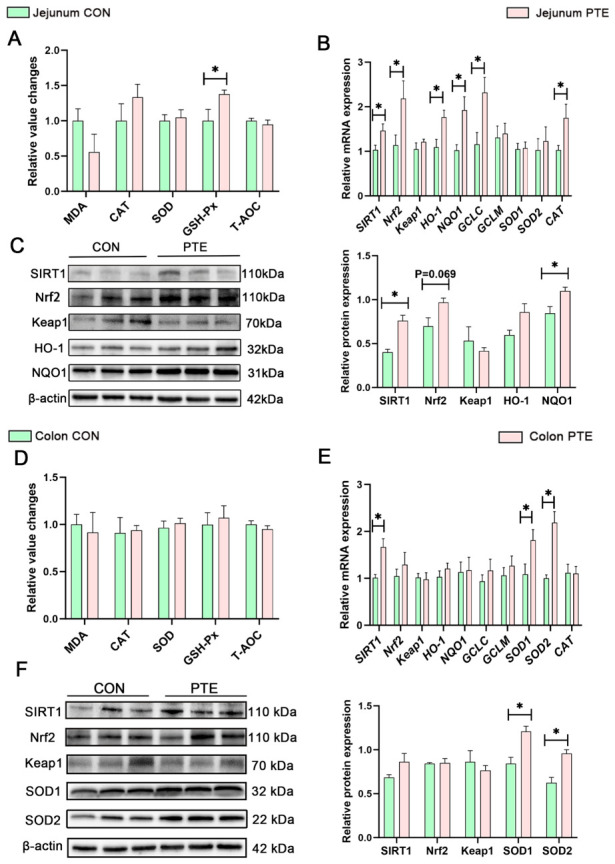
Effect of PTE on SIRT1-Nrf2/Keap1 and its antioxidant capacity in piglet intestine. (**A**) Antioxidant indication in the jejunum. (**B**) PTE on the expression of jejunal SIRT1-NRF2 axis and its downstream mRNA expression. (**C**) PTE on the expression of jejunal SIRT1-NRF2 axis and its downstream target proteins. (**D**) Antioxidant indication in the colon. (**E**) PTE on the expression of colonic SIRT1-Nrf2/Keap1 and its downstream mRNA expression. (**F**) PTE on the expression of colonic SIRT1-Nrf2/Keap1 and its downstream target proteins. CON: control group; PTE: pterostilbene group. Data are expressed as mean ± SEM (*n* = 6). * *p* < 0.05, compared with control group. The same β-actin loading control is shown for panels (**C**,**F**), as these targets were probed on the same membrane (see Section 2).

PTE significantly increased GSH-Px levels in the jejunum of piglets (Figure 8A, *p* < 0.05) but did not significantly affect various antioxidant indications in the colon of piglets (Figure 8D). The Nrf2/ARE signaling pathway, mediated by Nrf2, is a critical antioxidative defense pathway in body. Furthermore, SIRT1 promotes the deacetylation and nuclear translocation of Nrf2. Consequently, we explored whether PTE activates the SIRT1-Nrf2/Keap1 pathway and its downstream target genes within the gut. As shown in Figure 8B,C, the mRNA abundance and protein level of SIRT1 and NAD(P)H quinone dehydrogenase 1 (NQO1) in the jejunum of piglets in the PTE group were significantly higher (*p* < 0.05). Compared with the control group, Nrf2 nucleoprotein levels were numerically increased, although this difference did not reach statistical significance (*p* = 0.069). In addition, qRT-PCR results showed that the mRNA expression levels of glutamate-cysteine ligase catalytic subunit (*GCLC)* and catalase (*CAT)* downstream of the Nrf2 pathway were also significantly elevated in the jejunum of piglets in the PTE group (Figure 8B, *p* < 0.05). As shown in Figure 8E,F, the mRNA abundance and the protein amounts of SOD1 and SOD2 in the colon of piglets in the PTE group were significantly higher (*p* < 0.05). In addition, qRT-PCR results showed that the mRNA expression level of *SIRT1* was also significantly elevated in the colon of piglets in the PTE group (Figure 8E, *p* < 0.05), but the rest of the mRNA expression levels were not statistically significant.

### 3.9. Effects of PTE on Intestinal Lipid Metabolism and PI3K-AKT Signaling Pathway in Piglets

**Figure 9:**
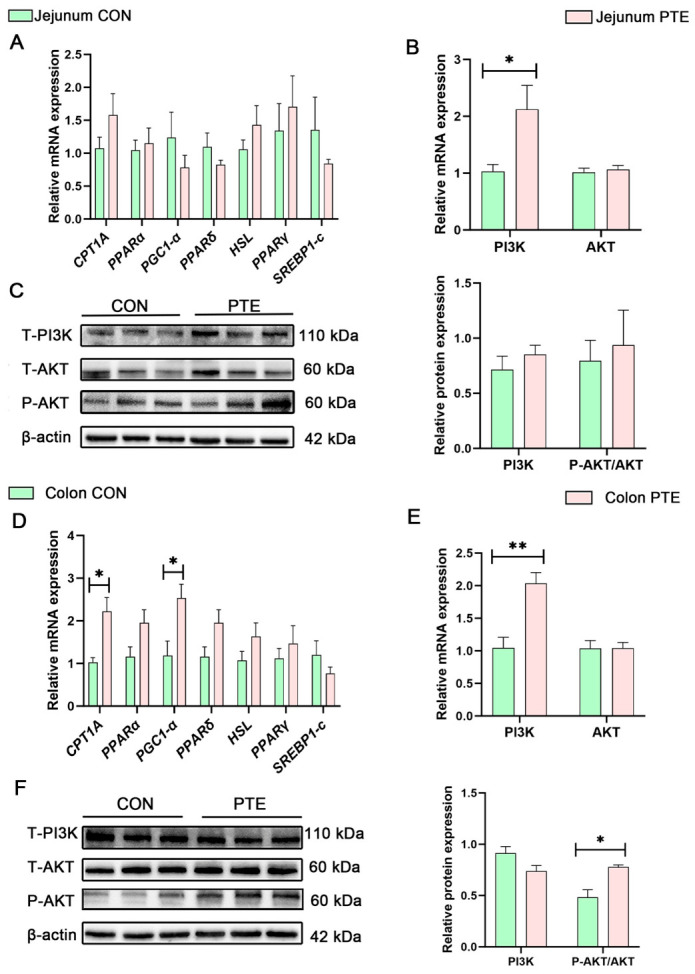
Effect of PTE on intestinal lipid metabolism and PI3K-AKT signaling pathway in piglets. (**A**) Expression of lipid metabolism mRNA in the jejunum. (**B**) Expression of PI3K-AKT signaling pathway mRNA in the jejunum. (**C**) Expression of PI3K-AKT signaling pathway proteins in the jejunum. (**D**) Expression of lipid metabolism mRNA in the colon. (**E**) Expression of PI3K-AKT signaling pathway mRNA in the colon. (**F**) Expression of PI3K-AKT signaling pathway proteins in the colon. CON: control group; PTE: pterostilbene group. Data are expressed as mean ± SEM (*n* = 6). * *p* < 0.05, ** *p* < 0.01, compared with control group. The same β-actin loading control is shown for panels C and F, as these targets were probed on the same membrane (see Section 2).

Changes in lipid metabolism-related genes in the jejunum were not statistically significant in the PTE group compared with the CON group (Figure 9A). In addition, the PI3K-AKT pathway is a key pathway in the regulation of energy metabolism. qRT-PCR analysis confirmed that the mRNA expression of phosphoinositide 3-kinase (*PI3K)* was significantly higher in the jejunum of piglets in the PTE group compared with the CON group (Figure 9B, *p* < 0.05). However, as shown in Figure 9C, Western blot analysis showed no significant changes in PI3K and P-AKT/T-AKT protein ratio in the jejunum of piglets in the PTE group. It is noteworthy that the metabolic regulatory characteristics of colonic tissues differ markedly from those of the jejunum. As shown in Figure 9D, compared with the CON group, the mRNA expression level of carnitine palmitoyltransferase 1A (*CPT1A)* and peroxisome proliferator-activated receptor gamma coactivator 1-alpha (*PGC1-α)* in the colon of piglets in the PTE group was significantly higher (*p* < 0.05). qRT-PCR analysis confirmed that the mRNA expression of *PI3K* in the piglet colon of the PTE group was markedly increased compared with that of the CON group (Figure 9E, *p* < 0.01). The results of the Western blot analysis revealed that the P-AKT/T-AKT protein ratio in the piglet colon of the PTE group was significantly higher (Figure 9F, *p* < 0.05). These findings indicate that PTE enhances fat β-oxidation through the activation of the colonic PI3K-AKT signaling pathway, thereby participating in the regulation of intestinal lipid metabolism.

### 3.10. Effect of PTE on the Colonic Mucosa Microbiota of Piglets

**Figure 10:**
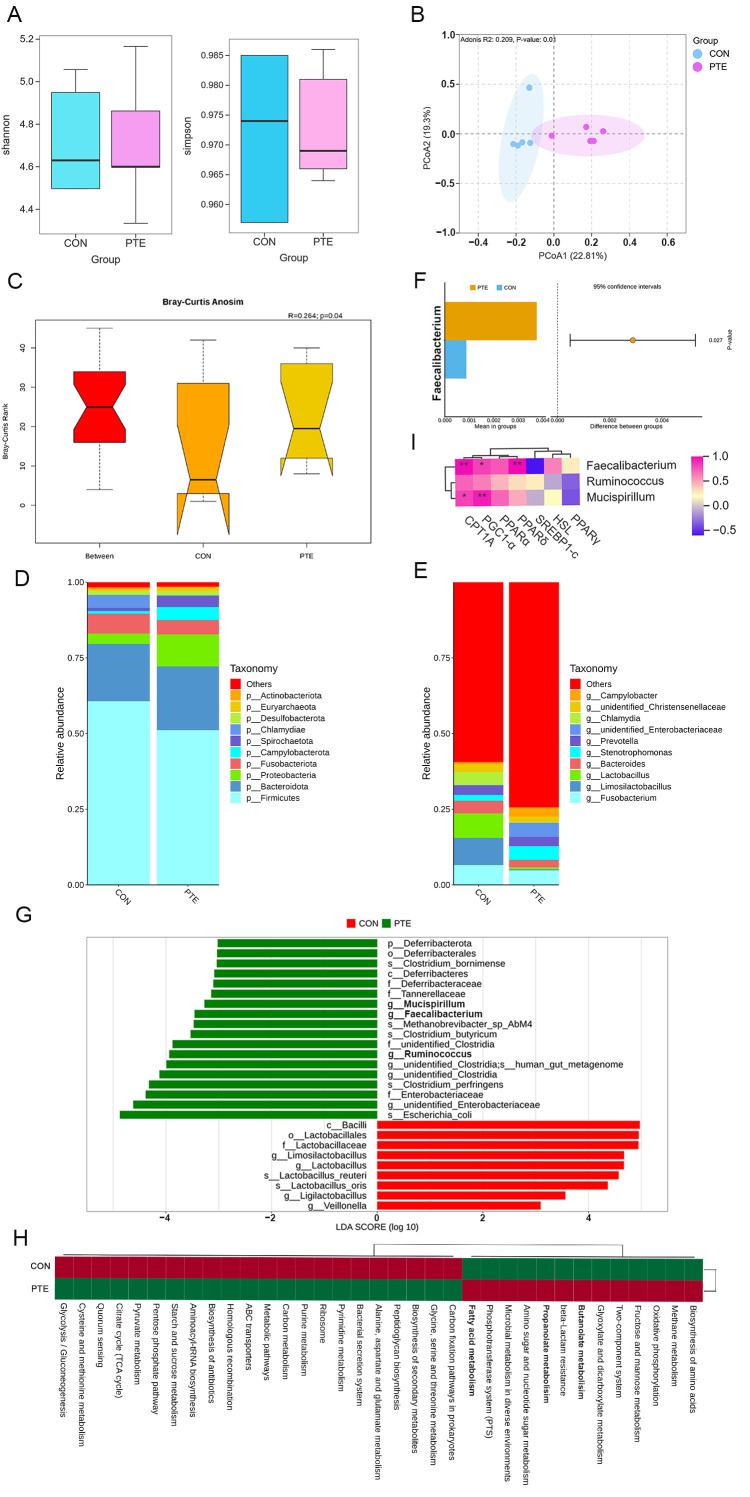
Effect of PTE on the colonic mucosa microbiota of piglets. (**A**) Colonic mucosal microbiota alpha diversity (Shannon and Simpson). (**B**) Colonic mucosal microbiota beta diversity. (**C**) Anosim analysis. (**D**,**E**) Relative abundance at the microbiota phylum and genus level. (**F**) *t*-test for microbial genus level of colonic mucosal microbiota. (**G**) LEfSe analysis. (**H**) Tax4Fun2 microbial function prediction. (**I**) Correlations between dominant bacterial genera and lipid metabolism genes based on Spearman’s correlation analysis. CON: control group; PTE: pterostilbene group. * *p* < 0.05 and ** *p* < 0.01, compared with control group (*n* = 5).

The formation of the intestinal mucosal layer is closely related to changes in the structural colonization of the intestinal microbiota. As shown in Figure 10A, although the addition of PTE in this study did not significantly affect the α diversity of the microbiota in the colonic mucosa of piglets, PCoA analysis based on binary_jaccard distance and Anosim test showed that there was a significant difference in the gut microbiota structure between the two groups of piglets (Figure 10B,C, R = 0.264, *p* < 0.05). The structure of the colonic mucosal microbiota of the two groups is broadly similar in composition at the phylum level, shown in Figure 10D, with Firmicutes (CON: 50.65%, PTE: 42.64%) and Bacteroidota (CON: 15.73%, PTE: 17.54%) as the dominant phyla. Figure 10E,F displays the two groups’ varying genus-level microbiota distributions. In comparison to the CON group, the PTE group had a considerably greater relative abundance of *Faecalibacterium* (CON: 0.00090 vs. PTE: 0.00377 *p* < 0.05). LEfSe analysis revealed that the dominant genera in the PTE group were *Mucispirllum, Faecalibacterium,* and *Ruminococcus* (Figure 10G, LDA > 3). The functional prediction of the colonic mucosa microbial community was performed using Tax4Fun2 (Figure 10H). It was found that the PTE group was mostly enriched in lipid metabolism-related pathways (fatty acid metabolism, butanoate metabolism and propanoate metabolism). Spearman’s correlation analysis between differential bacterial genera and lipid metabolism genes revealed significant positive correlations between *Faecalibacterium* and *CPT1A*, *PGC1-α*, and peroxisome proliferator-activated receptor delta (*PPARδ)*, and between *Mucispirillum* and *CPT1A* and *PGC1-α* (Figure 10I).

### 3.11. Effect of PTE on the Colonic Mucosa Metabolites of Piglets

**Figure 11:**
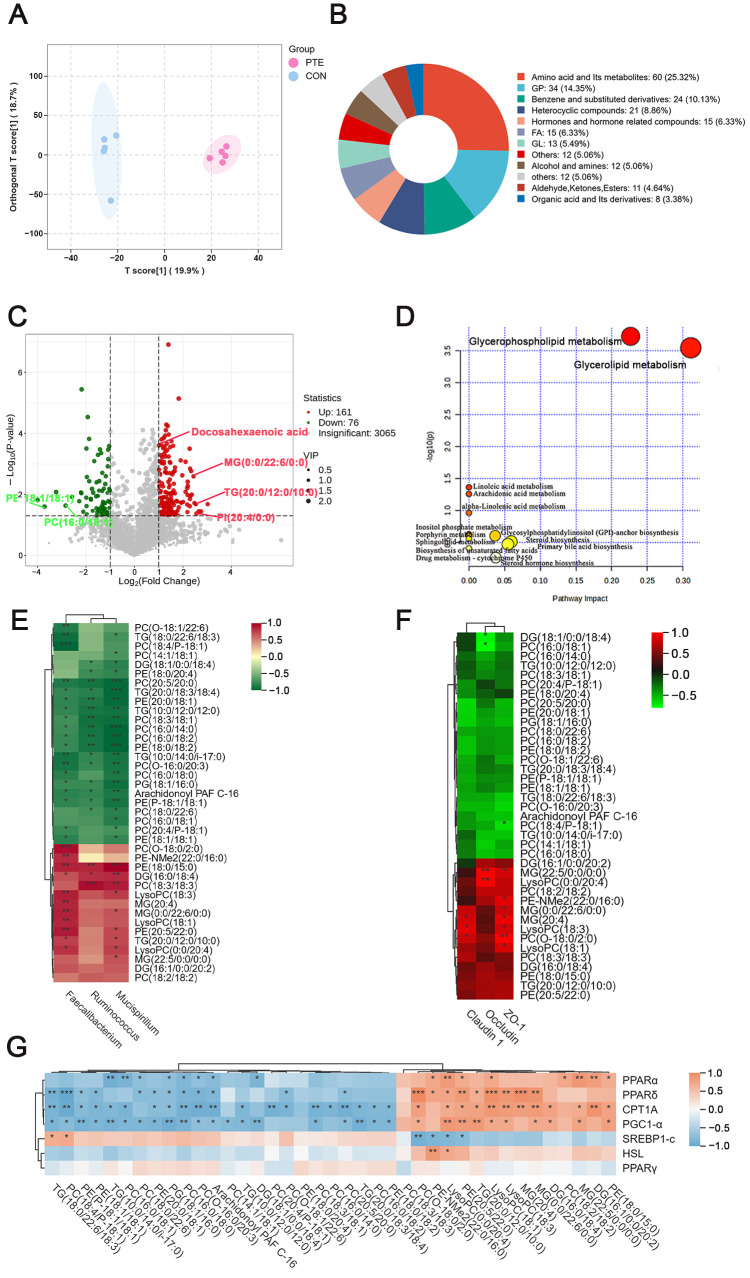
Effect of PTE on the colonic mucosa metabolites of piglets. (**A**) OPLS-DA analysis of metabolite composition. (**B**) Differential metabolite classification map. (**C**) Metabolite volcano plot (Red dots indicate significantly upregulated metabolites, and green dots indicate significantly downregulated metabolites). (**D**) Metabolic pathways according to the MetaboAnalyst analysis. (**E**) Correlations between different bacterial genera and different metabolites based on Spearman analysis. (**F**) Correlations between differential metabolites and genes related to gut barrier proteins based on Spearman’s correlation analysis. (**G**) Correlations between distinct differential metabolites and lipid metabolism genes based on Spearman’s correlation analysis. CON: control group; PTE: pterostilbene group. * *p* < 0.05, ** *p* < 0.01 and *** *p* < 0.001, compared with control group (*n* = 5).

Colonic mucosal metabolite levels were further examined using untargeted metabolomics. Detailed information on all metabolites identified in colonic mucosa is provided in Supplementary Table S6. The OPLS-DA data showed significant changes in colonic mucosal metabolites in the CON and PTE groups, and the same screening criteria were used to identify differential metabolites (Figure 11A). Differential metabolites were screened under the same conditions according to the OPLS-DA model. Among them, Figure 11B displays the top-five classes of primary DEMs: amino acid and its metabolites (25.32%), GP (14.35%), FA (6.33%), GL (5.49%) and organic acid and its metabolites (3.38%). The volcano plot showed a total of 237 significantly different metabolites between the two groups, of which 161 were upregulated and 76 were downregulated (Figure 11C). The top-five primary DEMs sorted by Log2FC values were predominantly GPs and GLs, including (MG (0:0/22:6/0:0), TG (20:0/12:0/10:0), PI (20:4/0:0), PE (18:1/18:1) and PC (16:0/18:1). It is noteworthy that docosahexaenoic acid (DHA) levels have increased significantly. MetaboAnalyst was usedto correlate metabolomics data with potential metabolic pathway regulation based on the 237 differential metabolites identified (Figure 11D). Fourteen metabolic pathways were enriched, which mainly contained 10 lipid metabolic pathways. Only two lipid metabolism pathways (glycerophospholipid metabolism and glycerolipid metabolism) remained significant after FDR correction (Benjamini–Hochberg method, q < 0.05). Spearman’s analysis revealed that PC and PE lipids (24/39) among the differentially enriched metabolites in these two key metabolic pathways exhibited a more pronounced correlation with the genus-level gut microbiota (Figure 10E). PC (O-18:1/22:6) and PC (18:4/P-18:1) showed significant negative correlations with *Faecalibacterium*. PC (16:0/18:0), PC (20:4/P-18:1), and PE (18:1/18:1) exhibited significant negative correlations with both *Faecalibacterium* and *Mucispirillum*. PC (14:1/18:1) showed a significant negative correlation with *Mucispirillum*. PE (18:0/20:4) and PC (18:0/22:6) exhibited significant negative correlations with *Ruminococcus* and *Mucispirillum*. PC (16:0/18:1) showed a significant negative correlation with *Mucispirillum*. PC (20:5/20:0), PC (18:3/18:1), PC (16:0/14:0), PC (16:0/18:2), PC (O-16:0/20:3), PC (16:0/18:0), Arachidonoyl PAF C-16, PE (20:0/18:1), PE (18:0/18:2), and PE (P-18:1/18:1) showed significant negative correlations with all differential bacterial genera. On the contrary, PC (O-18:0/2:0) and PE-NMe2 (22:0/16:0) showed significant positive correlations with *Faecalibacterium*. PC (18:3/18:3) showed significant positive correlations with *Ruminococcus* and *Mucispirillum*. PE (20:5/22:0) exhibited significant positive correlations with *Faecalibacterium* and *Mucispirillum*. PE (18:0/15:0) demonstrated significant positive correlations with all differential bacterial genera. Glycerolipid (10/39) also showed significant correlations. DG (18:1/0:0/18:4) exhibited a significant negative correlation with *Ruminococcus* and *Mucispirillum*. TG (18:0/22:6/18:3) showed a significant negative correlation with *Faecalibacterium* and *Mucispirillum*. TG (20:0/18:3/18:4), TG (10:0/12:0/12:0) and TG (10:0/14:0/i-17:0) exhibited significant negative correlations with all differential bacterial genera. Conversely, DG (16:0/18:4) showed significant positive correlations with *Ruminococcus* and *Mucispirillum*. MG (20:4) showed a significant positive correlation with *Faecalibacterium*. MG (22:5/0:0/0:0) demonstrated a significant positive correlation with *Mucispirillum*. Both MG (0:0/22:6/0:0) and TG (20:0/12:0/10:0) exhibited significant positive correlations with *Faecalibacterium* and *Mucispirillum*. Notably, TG levels decreased significantly (4/5), while MGs (3/3) containing long-chain polyunsaturated fatty acids (LC-PUFAs) all showed a marked increase. The further analysis of the correlations between differential metabolites and intestinal structural protein genes and lipid metabolism genes (Figure 11F,G) revealed that MG (22:5/0:0/0:0) was significantly positively correlated with *Occludin* and *ZO-1*. Concurrently, MG (20:4), PC (O-18:0/2:0) and LysoPC (18:3) exhibited significant positive correlations with *ZO-1* and *Claudin-1*. Additionally, the upregulated differential metabolites demonstrated significant positive correlations with the expression of lipid metabolism genes (mainly the fatty acid oxidation genes *CPT1A* and *PGC1-α*). In summary, maternal dietary supplementation with PTE alters intestinal lipid metabolism in offspring, primarily by reshaping the composition of intestinal metabolites through glycerolipid metabolism and glycerophospholipid metabolism.

### 3.12. The Role of PTE in Microbial-Metabolic Regulation of Colostrum, Mature Milk and Colonic Mucosa

**Figure 12:**
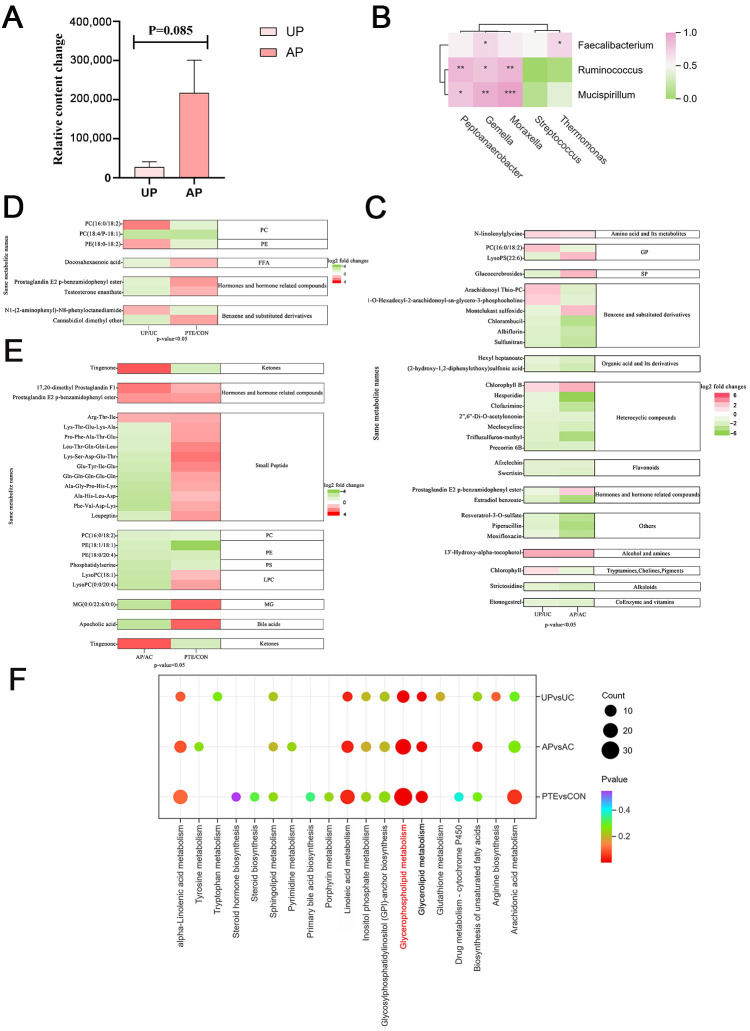
The role of PTE in microbial–metabolic regulation of colostrum, mature milk and colonic mucosa. (**A**) Levels of PTE in colostrum and mature milk. (**B**) Spearman correlation analysis of differential microbiota in colostrum, mature milk and colonic mucosa. (**C**) Same differential metabolites enriched in colostrum and mature milk. (**D**) Same differential metabolites enriched in colostrum and colonic mucosa. (**E**) Same differential metabolites enriched in mature milk and colonic mucosa. (**F**) Critical KEGG pathways enriched in colostrum, mature milk and colonic mucosa (Red highlights the only metabolic pathway that was significantly enriched in all three sample types (colostrum, mature milk, and colonic mucosa) after FDR correction). UC, AC, CON: control group; UP, AP, PTE: pterostilbene group. Data are expressed as mean ± SEM (*n* = 5). * *p* < 0.05, ** *p* < 0.01 and *** *p* < 0.001 compared with control group.

To determine the effect of maternal PTE supplementation on suckling piglets, we measured the relative content of PTE and its metabolites in colostrum, mature milk, and piglet colon. Results indicated the presence of PTE in both colostrum and mature milk, and, while the relative concentration in mature milk is higher than that in colostrum, the difference is not statistically significant (Figure 12A *p* = 0.085). However, neither PTE nor its typical Phase I (e.g., dihydropterostilbene, demethylpterostilbene) or Phase II (e.g., glucuronide, sulfate) metabolites were detected at levels reaching the lower limit of quantification in the colonic mucosa of the offspring. Therefore, the beneficial effects observed in suckling piglets in the PTE group are more likely to be achieved through indirect regulatory pathways mediated by breast milk. However, the specific mechanisms require further research to elucidate. The dynamic changes in infant colonic microbiota are closely associated with breast milk microbial regulation. Therefore, we further performed Spearman correlation analysis on differential microbiota in colostrum, mature milk, and colonic mucosa. The results revealed that, among the gut differential microbiota, only *Faecalibacterium* showed a significant positive correlation with colostrum-enriched *Thermomonas*. In contrast, all gut differential microbiota (*Faecalibacterium, Mucispirillum* and *Ruminococcus*) were positively correlated to varying degrees with the differential microbiota enriched in mature milk (Figure 12B). Enrichment analysis was performed on the differentially expressed metabolites identified in both colostrum and mature milk. Notably, this study revealed that PTE significantly increased the levels of N-linoleoylglycine, Chlorophyll B, and 13’-Hydroxy-alpha-tocopherol in both colostrum and mature milk (Figure 12C). The same differential metabolites identified in colostrum and colonic mucosa were summarized, and PC (18:4/P-18:1) levels were found to be significantly reduced in both samples (Figure 12D). We summarized the same differential metabolites identified in mature milk and colonic mucosa (Figure 12E). It is further observed that the levels of hormones and hormone-related compounds—17,20-dimethyl Prostaglandin F1 and Prostaglandin E2 p-benzamidophenyl ester—along with the small peptide Arg-Thr-Ile, were significantly elevated. Concurrently, we detected decreased levels of several glycerophospholipids: PC (16:0/18:2), PE (18:1/18:1), PE (18:0/20:4) and Phosphatidylserine. Furthermore, the analysis of all enriched metabolic pathways in colostrum, mature milk, and colonic mucosa revealed that lipid metabolism (including glycerophospholipid metabolism) may be a key metabolic pathway closely associated with milk-mediated maternal effects and offspring gut health (Figure 12F).

## 4. Discussion

PTE is known to have powerful antioxidant activity. Elevated levels of SOD and T-AOC in mature milk confirmed that PTE supplementation improved the antioxidant capacity of mature milk but had no significant effect on the antioxidant capacity of colostrum. A significant increase in polyphenol 2-vanillin in breast milk has been reported from 105.82 nmol/L in colostrum to 398.29 nmol/L in transitional milk [14]. In this study, the relative content of PTE in mature milk was higher than that in colostrum. Although the difference was not statistically significant, it still supported the dynamic changes in polyphenol levels in milk. In addition, it has been reported that animal experiments have confirmed that the antioxidant capacity of milk from sows fed resveratrol showed dynamic changes at different stages of lactation [15]. Changes in the content of total polyphenol extracts in breast milk at different lactation stages may be one of the key factors contributing to the stage-specific changes in its antioxidant capacity. It is worth noting that fluctuations in the levels of polyphenols in breast milk not only affect the redox state but may also be accompanied by a reshaping of the microenvironment of the milk, thereby potentially influencing the early establishment of the gut microbiota in newborn piglets.

Maternal dietary supplementation with polyphenols can promote the healthy colonization of beneficial bacteria within the infant’s gut by regulating the microbiota and its metabolites in breast milk [16]. This helps to maintain the redox balance and metabolic homeostasis of subsequent generations, exerting a long-term positive influence on their health. *Streptococcus* in colostrum plays a crucial role in establishing the microbiological barrier of the infant’s gut [17]. In addition, the present study found that the abundance of the dominant genus *Thermomonas* in the colostrum of the PTE group was significantly and positively correlated with SOD activity, and this finding may provide a new direction for the study of antioxidant mechanisms in breast milk. Although *Thermomonas* has been very limited in breast milk studies, it has been shown that its metabolic activity may contribute to optimizing the gut microenvironment [18]. Therefore, we reasonably infer that this microflora may be transmitted vertically from mother to offspring, thereby promoting the stability of their gut health. The predictive analysis of the functional composition of the colostrum microbiota revealed that PTE intervention primarily influences amino acid and lipid metabolism within colostrum. Metabolomic analysis corroborated this finding, further indicating a more pronounced effect on lipid metabolism. Although no significant enrichment was observed in amino acid metabolic pathways, it is noteworthy that we detected a marked elevation in levels of GSH, a potent antioxidant, within the milk [19]. In addition, the significantly elevated level of NAG, a derivative of glutamine, has been shown to partially prevent weight loss and maintain intestinal immune function in malnourished pigs [20]. This demonstrates that maternal dietary supplementation with PTE facilitates the accumulation of specific amino acid derivatives, thereby contributing to the optimization of functional components in milk. The lipid metabolism pathways significantly enriched in colostrum are glycerophospholipid metabolism and glycerolipid metabolism. It is important that all dominant bacterial genera induced by PTE in the colostrum were significantly negatively correlated with the glycerophospholipid metabolites LysoPA (18:0) and LysoPS (22:6). Lipopolysaccharide (LPS), as an endotoxin of Gram-negative bacteria, exacerbates inflammatory responses by activating TLR4, while lysophosphatidic acid (LPA) directly stimulates macrophages to release pro-inflammatory mediators such as interleukin-1 (IL-1) and reactive oxygen species (ROS) [21,22]. Therefore, the concurrent decrease in LPS and LPA may reflect a reduction in the inflammatory state within the breast. The glycerolipid metabolic pathway plays an important role in cellular metabolism and lactation maintenance [23]. DG (16:0/18:3), enriched in the colostrum glycerolipid metabolism, showed significant increase while MG (18:2/0:0/0:0) decreased markedly. It is worth noting that there is a significant positive association between DG (16:0/18:3) and SOD activity. Therefore, PTE supplementation may promote the formation of more stable glyceride structures in colostrum, thereby enhancing lipid oxidation stability. This synergistic effect may indicate potential health advantages for the offspring.

Compared with colostrum, mature milk harbors a relatively stable and persistent microbial community, which provides a continuous source of microorganisms and thereby facilitates the gradual maturation of the infant gut microbiota. PTE intervention significantly increased the abundances of *Gemella* and *Moraxella*, which are genera related to lipid metabolism, in mature milk [24,25]. Given that maternal dietary changes alter the breast milk microenvironment, this finding may not be coincidental. *Gemella* and *Moraxella*, though not traditionally regarded as core genera of exceptionally high abundance in milk, have been demonstrated to be stable core members of the milk microbiome [26,27]. Furthermore, metabolomics studies indicate that PTE significantly influences lipid metabolism in mature milk by regulating the abundance of microbial communities, particularly glycerophospholipid metabolism. Correlation analysis showed that LysoPCs and LysoPEs involved in glycerophospholipid metabolism were significantly negatively correlated with all different bacterial genera. ROS promote the hydrolysis of PC and PE by activating phospholipase A_2_, leading to elevated levels of LysoPC and LysoPE. These concentration changes can serve as markers of oxidative stress intensity [28]. Therefore, we hypothesized that PTE may attenuate oxidative damage to milk by inhibiting phospholipase activity. Meanwhile, dominant genera *Gemella* and *Moraxella* in mature milk were positively correlated with T-AOC, while LysoPC (18:1) and LysoPE (0:0/18:0) were negatively correlated with GSH-Px and T-AOC, respectively. This result coincided with the elevated multiple antioxidant indications of mature milk observed in the study, further validating the antioxidant protective effect of PTE. Similar studies have shown that dietary inulin supplementation reduces rumen fluid LysoPCs levels in dairy cows, which are negatively correlated with the quality of milk components [29]. Along with the concurrent increase in FFA (22:6), we observed positive correlations between DG (18:3/0:0/18:3), DG (18:3/18:2/0:0) and antioxidant indices. These results suggest that PTE may optimize the nutritional composition and oxidative stability of milk, thereby helping to enhance the antioxidant capacity of mature milk and potentially provide favorable conditions for the healthy development of offspring. However, the small multi-omics sample size (*n* = 5 per group) limits statistical power and downstream reliability, and therefore validation in larger-scale samples is needed.

The enhanced antioxidant activity in breast milk, combined with the role of the breast milk microbiota and its metabolites in shaping the offspring’s gut microenvironment, may help to improve the offspring’s gut morphology and have potential positive implications for their gut health. As key indicators of intestinal morphology, VH and CD directly reflect the absorption capacity of intestinal mucosa. The results of the present study showed that PTE significantly increased VC in the jejunum of piglets and improved the V/C, thus improving the intestinal morphology and structure. The observed improvement in intestinal morphology following nutritional intervention in our study is consistent with the findings reported by Mhalhel et al. [30]. Furthermore, that study appropriately noted the limitations associated with small-sample-size studies. Polyphenols may improve intestinal permeability by enhancing intestinal barrier function [31]; the present study also found that PTE enhanced the expression levels of key tight junction proteins (Claudin-1 and ZO-1) in piglet intestinal tissues, further validating this conclusion.

Improving intestinal barrier function helps to alleviate oxidative stress and maintain intestinal homeostasis. The Nrf2-ARE pathway is a major sensor and a major regulator of oxidative stress that regulates the expression of hundreds of antioxidant and detoxification genes [32]. Oxidative/electrophilic stress disrupts the cytoplasmic interaction between Keap1 and Nrf2, allowing Nrf2 to translocate to the nucleus. There, it forms heterodimers with small Maf proteins and binds to antioxidant response elements (AREs), thereby initiating the transcription of heme oxygenase-1 (HO-1), NQO1, GCLC, and glutamate-cysteine ligase modifier subunit (GCLM) [33]. Given that GCLC and GCLM constitute the rate-limiting enzymes for intracellular GSH synthesis [34], the concurrent elevation of jejunal GSH levels we observed strongly suggests this may result from the activation of the Nrf2 pathway. Notably, although the increase in Nrf2 protein levels did not reach statistical significance, SIRT1, which is a known upstream activator of Nrf2, was significantly upregulated, and the protein expressions of NQO1 and HO-1, which are the downstream targets of Nrf2, also significantly increased. However, these changes did not significantly modulate the Nrf2 pathway in the piglet colon. Recently, in vitro and in vivo studies have identified PTE as a potent Nrf2 activator [35,36]. In addition, SIRT1 can promote Nrf2 nuclear translocation, and it is noteworthy that the activation of the Keap1-Nrf2 pathway is closely associated with increased SIRT1 protein expression [37]. The key point is that intestinal development in suckling piglets centers on the small intestine. The jejunum serves as the primary site for digesting and absorbing key nutrients from breast milk. Consequently, jejunal cells may encounter higher concentrations of PTE and its early metabolites during this absorption process, thereby exerting a more potent regulatory effect on the Nrf2 pathway.

Polyphenols effectively maintain the balance of lipid metabolism and enhance antioxidant defense by regulating the interaction between oxidative stress and nutrient-sensing signaling pathways [38,39]. It has been reported that maternal polyphenol supplementation (including RSV) during fetal development and lactation exerts beneficial metabolic programming effects [40]. Lipid metabolism involves multiple physiological processes, including lipolysis, fatty acid oxidation, lipogenesis, and fat deposition, and is regulated by key transcription factors. CPT1A and peroxisome proliferator-activated receptor alpha (PPARα) play critical roles in regulating fatty acid oxidation [41]. PGC-1α, as a transcriptional coactivator, cooperates with PPARδ to regulate fatty acid oxidation processes [42]. Hormone-sensitive lipase (HSL) is a rate-limiting enzyme in lipolysis that primarily catalyzes the hydrolysis of stored TGs into free fatty acids and glycerol in adipose tissue [43]. peroxisome proliferator-activated receptor gamma (PPARγ) and sterol regulatory element-binding protein 1c (SREBP-1c) are transcription factors associated with lipid synthesis [44]. The present study found that the addition of PTE exhibited a upregulation in the mRNA expression of genes associated with fatty acid β-oxidation (*CPT1A* and *PGC1-α*) in the colon. However, it had no significant effect on the expression of genes related to lipolysis (such as *HSL*) or lipid synthesis (such as *SREBP-1c* and *PPARγ*). Shimoda et al. also reported that polyphenol-rich walnut extract reduces triglyceride levels by enhancing peroxisomal fatty acid β-oxidation [45]. The PI3K signaling pathway plays a pivotal role in regulating cellular energy metabolism through its downstream effector AKT. It has been reported that Cr(III) promotes lipolysis by activating the PI3K/AKT signaling pathway, which enhances the nuclear level of PPARα and the expression of its downstream fat oxidation genes [46]. Importantly, PTE can exert a positive effect on intestinal lipid metabolism indirectly by promoting a healthy microbial environment [11]. These data support the possibility that PTE optimizes lipid metabolic homeostasis by activating the colonic PI3K-AKT signaling pathway and synergizing with fatty acid oxidation processes.

There is a close association between the composition of the gut microbiota and its regulatory role in lipid metabolism signaling pathways [47]. In the present study, it was found that the dominant genera of PTE-induced colonic mucosa were *Mucispirillum*, *Faecalibacteriumin* and *Ruminococcus*. *Mucospirillum* is a mucosa-colonizing commensal bacterium that competitively inhibits pathogen adhesion by forming protective biofilms, thereby enhancing the host’s mucosal innate defense [48]. *Ruminococcus* and *Faecalibacterium* are the primary butyrate producers in the gut [49]. Butyrate serves as the primary energy source for colonic cells, promoting cellular health and function [50]. However, subsequent studies should further measure the Short-Chain Fatty Acids (SCFAs) concentration in colonic contents to validate this finding. Notably, the microbial functional prediction results for the PTE group’s colonic mucosa align with metabolomics findings, collectively indicating that the microbiota and its metabolites exert regulatory roles in intestinal lipid metabolism pathways. Metabolomics further identified glycerophospholipid metabolism and glyceride metabolism as core pathways regulating intestinal metabolites. Glycerophospholipids, such as phosphatidylcholine, are crucial for cell membrane integrity and serve as carriers for essential nutrients like choline and n-3 PUFAs [51]. Different types of phospholipids possess unique physiological functions. Joint analysis revealed significant correlations between dominant gut bacterial genera and colonic mucosal PC and PE levels. Interestingly, the relative content of phosphatidylcholine and phosphatidylethanolamine containing n-6 PUFAs (particularly arachidonic acid) decreased, with representative metabolites including PE (18:0/20:4) and PC (20:4/P-18:1). Arachidonic acid, as a precursor to eicosanoids, is well-known to mediate inflammatory responses [52]. Crucially, we observed an accumulation of phospholipids containing n-3 PUFAs, including PC (18:3/18:3) and PE (20:5/22:0). Research indicates that high n-6 PUFA content correlates with heightened inflammatory responses and oxidative stress. Conversely, n-3 PUFAs are linked to the production of anti-inflammatory mediators that can reduce inflammation and promote remission [53]. Furthermore, correlation analysis revealed that PC (O-18:0/2:0) positively correlated with the gene expression of intestinal structural proteins Claudin-1 and ZO-1. Ether-linked phospholipids possess antioxidant activity due to their more stable chemical structure, which may contribute to enhancing intestinal barrier stability [54]. Meanwhile, the glycerolipid profile of intestinal metabolites was modified, with decreased overall levels of TGs (4/5) and increased levels of LCFA-MAG (3/3), including MG (22:6) and MG (22:5) associated with DHA and its precursors. In addition, the relative level of DHA itself was also upregulated. These observations are consistent with enhanced fatty acid oxidation, and suggest that n-3 polyunsaturated fatty acid metabolism may be in an active state. Consequently, PTE may reshape the gut microbial composition and alter its metabolites, thereby contributing to the maintenance of intestinal lipid metabolic homeostasis. This process may create favorable conditions for the activation of the PI3K-AKT pathway and provide support for the enhancement of intestinal barrier integrity, ultimately exerting a positive regulatory effect on overall intestinal physiological function. Nevertheless, the causal relationship and mechanisms between the microbiome and metabolome warrant further investigation and exploration.

The breast milk microbiota plays a crucial role in shaping the infant gut microbiota, thereby influencing health outcomes during early life [55]. This study found that the microbial community in mature milk has a significantly stronger influence on the gut microbiota of piglets than colostrum, and that the cumulative effect of PTE is more pronounced in mature milk than in colostrum. This suggests that PTE may influence the early colonization and development of the offspring’s gut microbiota by modulating the dynamic composition of the breast milk microbiota. Intriguingly, PTE increased levels of two bioactive lipids: N-linoleoylglycine, an anti-inflammatory mediator [56], and 13′-hydroxy-α-tocopherol, a precursor of a vitamin E metabolite known to modulate antioxidant enzymes [57]. This dual increase further supports the idea that PTE enhances the functional, anti-inflammatory, and antioxidant quality of both colostrum and mature milk. Furthermore, pathway enrichment analysis indicates that breast milk and the offspring’s gut lipid metabolism pathways share consistent enrichment patterns. This suggests that PTE may influence milk composition by regulating lipid metabolism, thereby indirectly modulating lipid metabolism in the offspring’s gut and creating favorable conditions for gut health and normal growth and development. Similarly, due to the small multi-omics sample size (*n* = 5 per group), these findings require validation in larger cohort studies and further refinement through more in-depth mechanistic investigations.

## 5. Conclusions

In conclusion, after supplementing 500 mg/kg PTE, the microbiota and metabolites in the breast milk changed. These changes may be the key to enhancing the antioxidant capacity of breast milk and improving the redox balance in the jejunum of piglets. Moreover, PTE reshaped the colonic microbiota structure of piglets. Meanwhile, both piglet colonic metabolites and breast milk metabolites were enriched in the glycerophospholipid metabolism. This convergence suggests that PTE-induced shifts in glycerophospholipid metabolism may represent a shared metabolic feature linking milk composition to piglet colonic development. This study provides new insights for the further development and application of PTE as a functional food ingredient.

## Figures and Tables

**Table 1:** Effect of PTE on piglets’ performances.

Item ^1^	Treatments		SEM	*p*-Value
	CON	PTE		
Total live litter weight at birth, kg	17.22	16.32	1.132	0.511
Average live weight at birth, kg	1.44	1.42	0.114	0.883
Total litter weight at weaning, kg	53.54	63.09	5.667	0.123
Average weight at weaning, kg	5.96	6.25	0.275	0.322
Litter weight gain, kg	36.32	46.76	5.666	0.095
ADG, kg/d	0.22	0.23	0.013	0.285

^1^ Effect of PTE on piglet production performance. Note: Average daily gain (ADG) = (weaning weight − birth weight)/days; CON = control group; PTE = pterostilbene group. Data are expressed as mean ± SEM (*n* = 6).

## Data Availability

The original contributions presented in this study are included in the article/Supplementary Materials. Further inquiries can be directed to the corresponding authors.

## Supplementary Materials

The following supporting information can be downloaded at: https://www.mdpi.com/article/10.3390/antiox15050531/s1. Table S1. Ingredient composition and nutritional levels of the basal diet (air-dried basis, %); Table S2. Primers and PCR products for gene expression analysis by Real-Time qPCR; Table S3. Information for primary and secondary antibodies; Table S4. Colostrum all metabolite information; Table S5. Mature milk all metabolite information; Table S6. Colonic mucosa all metabolite information.

## Abbreviations

The following abbreviations are used in this manuscript:

AC	Mature milk control group
AKT	Protein kinase B
AP	Mature milk pterostilbene group
CAT	Catalase
CON	Control group
CPT1A	Carnitine Palmitoyltransferase 1a
GCLC	Glutamate-Cysteine Ligase Catalytic Subunit
GCLM	Glutamate-Cysteine Ligase Modifier Subunit
GSH-Px	Glutathione peroxidase
HO-1	Heme Oxygenase-1
HSL	Hormone-sensitive lipase
IF	Immunofluorescence
Keap1	Kelch-like ECH-associated Protein 1
MDA	Malondialdehyde
NQO1	NAD(P)H quinone oxidoreductase 1
Nrf2	Nuclear factor erythroid 2-related factor 2
PGC1-α	Peroxisome Proliferator-Activated Receptor Gamma Coactivator 1-Alpha
PI3K	Phosphatidylinositol-3-kinase
PPARα	Peroxisome Proliferator-Activated Receptor Alpha
PPARδ	Peroxisome Proliferator-Activated Receptor Delta
PPARγ	Peroxisome Proliferator-Activated Receptor Gamma
SIRT1	Sirtuin 1
SOD	Superoxide dismutase
SOD1	Superoxide dismutase 1
SOD2	Superoxide dismutase 2
SREBP1-c	Sterol Regulatory Element-Binding Protein 1c
T-AOC	Total antioxidative capacity
