# Supplementary material for: Effects of Maternal Pterostilbene Supplementation on Milk Composition and Offspring Gut Antioxidant/Lipid Metabolism in Suckling Piglets: A Multi-Omics Study
Source: Antioxidants (Basel). 2026 Apr 23;15(5):531. doi: 10.3390/antiox15050531 (PMC13203149; doi:10.3390/antiox15050531)
Supplement: Supplementary file 1 [file antioxidants-15-00531-s001.zip › Supplementary Table S1-3.pdf]

Supplementary Table S1 Ingredient composition and nutritional levels of the basal diet (air-dried basis, %)

Item (%)	Lactation diet
Ingredients	
Corn	580
Corn germ meal	40
Soybean meal, de-hulled	165
Rice bran meal	130
Soybean oil	20
Sodium chloride	4.0
Choline (50%)	2
Soya bean hull	20
CaHPO4	10
Lysine (80%)	8
Tryptophan (98.5%)	0.5
Threonine (98.5%)	0.5
Limestone	15
Premix[1]	5.0
Analyzed nutrient levels[2]	
CF	5.62
CP	17.17
Calculated energy and nutrient level[3]	
Net energy	2.3
Digestible energy (Mcal/kg)	3.29
OM	94.45
Lysine	1.25
Leucine	1.73
Methionine	0.44
Tyrosine	0.78
Threonine	0.81
Tryptophan	0.28
Na	0.38
Calcium	0.96
Cl	0.38
Total phosphorus	0.86
Available phosphorus	0.40

[1] The premixes provide per kilogram of diet: 100 mg Zn; 140 mg Vitamin E; 0.3 mg Se; 8,000 lU Vitamin A; 80 mg Fe; 2 mg Vitamin K3; 25 mg Mn; 2.4 mg Vitamin B1; 8.5 mg Vitamin B2; 20 mg Cu; 4.5 mg Vitamin B6; 0.03 mg Vitamin B12; 20 mg pantothenic acid; 3.5 mg folic acid; 0.4 mg I; 28 mg biotin; 28 mg niacin; 2,000 IU Vitamin D3. The source and element content of zinc, copper, iron, manganese, iodine and selenium were ZnSO4 (⩾34.5%), CuSO4(⩾25.0%), FeSO4 (⩾30.0%), MnSO4 (231.8%), CaI2 (⩾1.5%), NaSeO(⩾2.2%).

[2] The crude fiber (CF) content was determined according to GB/T 6434-2022, while crude protein (CP) was calculated as N x 6.25 using the Kjeldahl method (GB/T 6432-2018) with a FOSS

Kjeltec 8400 analyzer.
[3] Energy and nutrient level were calculated value.

Supplementary Table S2 Primers and PCR products for gene expression analysis by Real-Time qPCR

Gene	Primer sequence (5'-3')	Fragments sizes (bp)	Accession NO.
β-actin	F: GGCACCACACCTTCTACAACGAG	102	XM_003124280.5
	R: TCATCTTCTCACGGTTGGCTTTGG		
HSL	F: TTGAAATGCCACTGACTGCTGAC	132	NM_214315.3
	R: GCTCCTCACTGTCCTGTCCTTC		
PI3K	F: GGCAATGTGGAGCAGATGAAGG	109	XM_021102206.1
	R: GGTAGAGCAGGAGGAAGTGGTC		
AKT	F: GGCAATGTGGAGCAGATGAAGG	124	XM_021081501.1
	R: GGTAGAGCAGGAGGAAGTGGTC		
Nrf2	F: CCAATTCAGCCAGCACAACACATC	149	XM_013984303.2
	R: GACTGAGCCTGGTTAGGAGCAATG		
Keap1	F: GGAGGACCACACCAAGCAAGC	142	NM_001114671.1
	R: GGATGAAGCCAGCACCACCTTG		
SIRT1	F: GGCAGTAACAGTGAGAGTGGAAC	120	NM_001145750
	R: TCGCATATTAACATCAGCATCATCTTC		
HO-1	F: GTTTGAGGAGGTGCAGGAGC	184	NM_001004027.1
	R: GAGTGTCAGGACCCATCGGA		
NQO1	F: AGTATCCTGCCGAGACTGCTCTG	95	NM_001159613.1
	R: CACAAGGTCTGCGGCTTCCAC		
SOD1	F: CCAGTGCAGGTCCTCACTTCAATC	172	NM_001190422.1
	R: CGGCCAATGATGGAATGGTCTCC		

SOD2	F: TGTATCCGTCGGCGTCCAAGG	93	NM_214127.2
	R: TCCTGGTTAGAACAAGCGGCAATC		
GCLM	F： CACAGCGAGGAGCTTCGAGAC	119	XM_001926378.4
	R: ACTGCGTGAGACACAGTACATTCC		
GCLC	F: GCATGTGGCTCACCTCTTCATCAG	135	XM_021098556.1
	R: GGAGGCTTGAATCTCATCGTCTGC		
CAT	F: AGCCTACGTCCTGAGTCTCTGC	90	NM_214301.2
	R: TCCATATCCGTTCATGTGCCTGTG		
Occludin	F: CCTCCTCCCCTTTCGGACTA	70	NM_001163647.2
	R: TCACTTTCCCGTTGGACGAG		
ZO-1	F: TCAAGGTCTGCCGAGACAAC	140	XM_021098896.1
	R: ATCACAGTGTGGTAAGCGCA		
Claudin1	F: ATGACCCCAGTCAATGCCAG	95	NM_001244539.1
	R: CAAAGTAGGGCACCTCCCAG		
PPARα	F: AATAACCCGCCTTTCGTCATACAC	106	NM_001044526.1
	R: CCTCCGCCTCCTTGTTCTGG		
PPARδ	F: CAAGGCATCAGGCTTCCACTAC	104	NM_001130241.3
	R: TCCGCTCACACTTCTCGTACTC		
PPARγ	F: TCTGTGGACCTGTCGGTGATG	103	NM_214379.1
	R: TGTCTTCATAGTGTGGAGTGGAAATG		
SREBP1-c	F: AGCCAGATGAAGCCAGAGCAG	89	NM_214157.1
	R: AGGAAGACGAGCACGCACAG		
PGC1-α	F: GTTTGATGACAGCGAAGATGAAAGTG	97	NM_213963.2
	R: AGAAGAACAAGAAGGCGACACATC		

Supplementary Table S3 Information for primary and secondary antibodies

Primary antibodies	Dilution ratio	Source of antibodies	Cat.NO.	Clone type
Rabbit Anti-PI3K P110β antibody	1:1000	Wanleibio, Shenyang, China	WL03380	Polyclonal antibody
Rabbit Anti-Phospho-Akt antibody	1:500	ABclonal, Wuhan, China	AP1208	Polyclonal antibody
Rabbit Anti-Pan-Akt antibody	1:500	ABclonal, Wuhan, China	A18675	Polyclonal antibody
β-actin Rabbit mAb	1:5000	ABclonal, Wuhan, China	WH233691	Polyclonal antibody
SOD1 Rabbit mAb	1:1000	ABclonal, Wuhan, China	A12537	Polyclonal antibody
SOD2 Rabbit mAb	1:500	ABclonal, Wuhan, China	A21805	Polyclonal antibody
Heme Oxygenase 1 (HO-1/HMOX1) Rabbit pAb	1：1000	ABclonal, Wuhan, China	A1346	Polyclonal antibody
NRF2 Rabbit pAb	1:500	ABclonal, Wuhan, China	A0674	Polyclonal antibody
KEAP1 Rabbit pAb	1:100	ABclonal, Wuhan, China	A1820	Polyclonal antibody
Anti-SIRT1 Rabbit pAb	1:500	Wanleibio, Shenyang, China	WL02995	Polyclonal antibody
Secondary antibody	Dilution ratio	Source of antibodies	Cat.NO.	
HRP Goat Anti-Rabbit IgG (H+L)	1:3000	Servicebio, Wuhan, China	GB23303	
